# Integrated bioinformatics analysis identified MTHFD1L as a potential biomarker and correlated with immune infiltrates in hepatocellular carcinoma

**DOI:** 10.1042/BSR20202063

**Published:** 2021-02-19

**Authors:** Junhui Chen, Jie Yang, Qingchun Xu, Zhenyu Wang, Jun Wu, Liukui Pan, Kai Huang, Chen Wang

**Affiliations:** Emergency Department, The Second People’s Hospital of Wuhu, No. 259, Jiuhua Central Road, Wuhu, Anhui Province 241000, China

**Keywords:** hepatocellular carcinoma, MTHFD1L, tumor microenvironment, immune cell, prognosis

## Abstract

Liver hepatocellular carcinoma (LIHC) is one of the most frequently occurring primary malignant liver tumors and seriously harms people’s health in the world. Methylenetetrahydrofolate dehydrogenase 1-like (MTHFD1L) has been shown to be associated with colon cancer cell proliferation, colony formation and invasion. In the present study, a total of 370 LIHC and 51 normal samples data were downloaded from The Cancer Genome Atlas (TCGA) database. Bioinformatics and immunohistochemistry (IHC) analysis showed that MTHFD1L is highly expressed in liver tumors. Correlation analysis suggested the differences of vital status between high- and low-expression MTHFD1L groups of LIHC. Univariate and multivariate Cox proportional hazards regression were performed to identify the relationship between clinical characteristics and overall survival (OS). In addition, to explore whether MTHFD1L has an effect on the immune infiltration of LIHC. The correlation between MTHFD1L expression and 24 immune cells were analyzed by ImmuneCellAI database. Furthermore, we combined three databases CIBERSORT, TIMER and ImmuneCellAI to do a comprehensive validation and determined that dendritic cells (DCs) resting, macrophage M0 and macrophage M2 closely related to the expression of MTHFD1L. The results showed that MTHFD1L was a potential prognostic biomarker for LIHC, and could help to elucidate that how the immune microenvironment promotes liver cancer development.

## Introduction

Biomarkers are urgently needed for the diagnosis of cancers, such as gastric cancer [1] and liver cancer [2]. Primary liver cancer is one of the most common cause of cancer-related deaths, and is one of the few neoplasms with a steady increasing incidence and mortality in the world [3–5]. There are several main risk factors for liver cancer include excessive alcohol consumption and smoking tobacco, aflatoxin exposure, infection with hepatitis B or C, obesity and diabetes mellitus [3,6–9]. Liver cancer also includes liver hepatocellular carcinoma (LIHC), cholangiocarcinoma and mixed hepatocellular carcinoma, in which LIHC accounts for above 90% of liver cancers [3,10,11]. The prognosis of LIHC depends on the tumor stage at the time of diagnosis, and early detection of liver cancer is easier to obtain treatment options [12].

Therefore, it is urgent to find new and effective biomarkers for LIHC to improve clinical efficacy. Methylenetetrahydrofolate dehydrogenase 1-like (MTHFD1L), is an enzyme involved in tetrahydrofolate (THF) synthesis in mitochondria [13], participates in folic acid cycle and to form formate [14]. Folic acid disorders can contribute to predisposition to some diseases, including immune dysfunction and cancer [15]. Despite the importance of MTHFD1L in the folic acid cycle, knowledge about its role in human diseases, especially cancer, is still largely unknown. A recent study reported that MTHFD1L plays an important role in bladder cancer cell proliferation, colony formation and invasion, which was related to the prognosis [16]. Another study reported that MTHFD1L was involved in colorectal cancer progression, and blocking MTHFD1L reduces the growth of colon cancer cells, thus providing an avenue to target this enzyme with small molecule inhibitors [14]. However, the potential prognostic value of MTHFD1L and its impact on the immune infiltration of LIHC tumors are still unclear.

Plenty of studies have confirmed that the tumor microenvironment (TME) plays a vital role in the occurrence, development and progression of tumors [17–19]. TME refers to the environment surrounding the tumor, including surrounding blood vessels, tumor-infiltrating immune cells (TIICs), fibroblasts, signaling molecules and extracellular matrix [20,21]. TIICs play an important role in the TME and their activation status is different in TME that affects the therapeutic effect of tumor [20]. In the malignant cells inside the tumor, the abundance of TIICs has different degrees of heterogeneity due to the different TMEs, enhancing the immune cell function of tumors is still the main challenge of cancer therapy [22,23].

In this work, we analyzed the RNA-Seq and clinical data of 370 LIHC patients from The Cancer Genome Atlas (TCGA), to identify the relationship between MTHFD1L and clinical information, the expression level of MTHFD1L in liver cancer was verified by Oncomine database and Immunohistochemistry (IHC) study. Furthermore, the data on immune system infiltrates of LIHC from TCGA database were analyzed through the ImmuneCellAI, CIBERSORT and TIMER. The identification of prognostic MTHFD1L suggests the potential role of MTHFD1L in LIHC pathogenesis and progression.

## Materials and methods

### Raw data acquisition

A total of 370 LIHC patients and 51 control samples data were downloaded from the TCGA database (https://portal.gdc.cancer.gov/). Thirty-five tumor and 10 control samples were obtained from Oncomine (http://www.oncomine.org) database [24], a web‐based data mining platform, harbored the microarray databases of most human cancers. Some samples with incomplete clinical information were deleted when LIHC samples were analyzed by comprehensive bioinformatics. A total of 366 patients with RNA sequencing data and clinical information was obtained from TCGA database. The statistical difference of these features between high- and low-expression MTHFD1L groups was evaluated using chi-square test.

### Assessment of prognostic clinical characteristics and MTHFD1L in LIHC cohort

According to the level of MTHFD1L expression, we divided 366 LIHC samples into high-expression group and low-expression group, and chi-square test was used to evaluate the correlation between MTHFD1L expression level and clinical characteristics. Then we performed Kaplan–Meier analysis on 366 liver cancer patients through SPSS software (version 24.0, Chicago, IL, United States). A univariate Cox proportional hazard regression analysis was used to evaluate the association between overall survival (OS) time and clinical characteristics in the test cohort. The prognostic value of was considered statistically significant as the *P*-value was <0.05. Subsequently, a multivariate Cox proportional hazards regression analysis was performed to determine the clinical features than can be used as prognosis.

### Correlation analysis between MTHFD1L and immune infiltration in LIHC

The composition and abundance of immune cells in the TME have a great influence on tumor progression and the effect of immunotherapy. GraphPad Prism software (version 8.4.2, https://www.graphpad.com/updates/) was used to analyze the correlation between MTHFD1L expression and TIICs, the cutoff was *P*-value <0.01. The Immune Cell Abundance Identifier (ImmuCellAI) detected the abundance of immune cells, and the differences in the infiltration immune cells of 366 LIHC were analyzed. ImmuneCellAI is a tool for estimating the abundance of 24 immune cells from gene expression datasets, including RNA-Seq and microarray data, of which 24 immune cells are composed of 6 immune cell types and 18 T-cell subtypes (http://bioinfo.life.hust.edu.cn/web/ImmuCellAI/) [25]. The TIMER (https://cistrome.shinyapps.io/timer/) [26] and CIBERSORT (http://cibersort.stanford.edu/) [27] were used to evaluate the results of tumor immune infiltration analysis in the ImmuneCellAI database.

### IHC

The protein levels of MTHFD1L (Abcam) in formalin-fixed paraffin embedded liver cancer tissues and adjacent non-tumor tissues were detected by IHC. Briefly, the sections were incubated with primary antibody and secondary antibody, visualized with 3,3′-diaminobenzidine and then counterstained with Hematoxylin for examination by the microscope. Fifteen liver cancer tissues and the paired adjacent normal tissues were chosen randomly. The sections were photographed under a microscope and at least six fields were examined.

### Statistical analysis

All statistical analyses were enforced with corresponding packages of R (Version 3.6.3) and analyzed using SPSS (version 24.0). Kaplan–Meier survival analysis and chi square test was conducted using SPSS (version 24.0). To calculate the 95% CI and HR, we used both the univariate and multivariate models of the Cox analysis. Univariate and multivariate Cox regression analyses were used to evaluate MTHFD1L expression between clinical factors and OS. For all analysis, the threshold *P*-value <0.05 was considered significant statistically.

## Results

### MTHFD1L had high expression in LIHC

**Figure 1:**
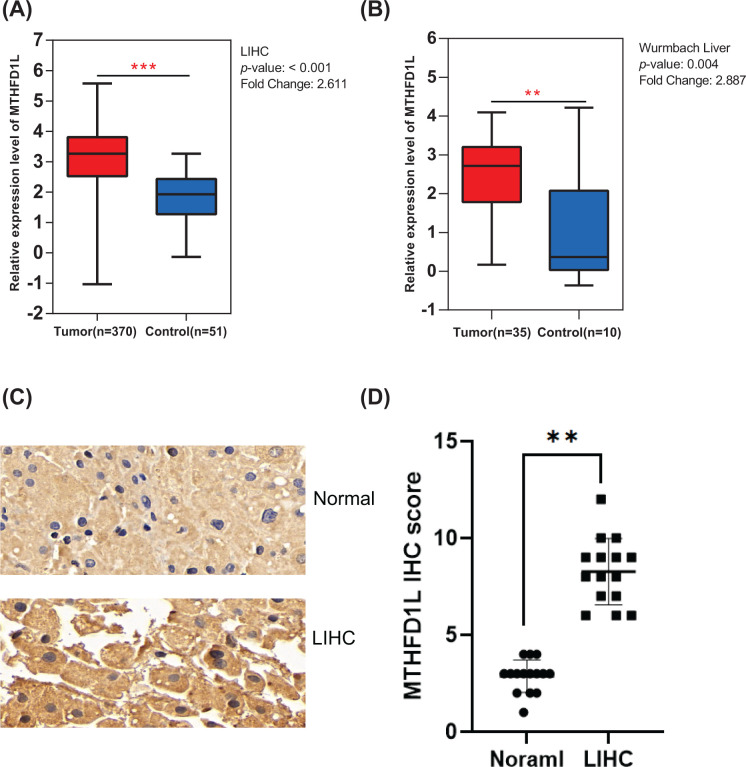
The expression of MTHFD1L in tumor and normal tissues (**A**) Expression levels of MTHFD1L in LIHC and normal tissue detected by TCGA database (*P*-value <0.001, FC = 2.611); (**B**) The expression of MTHFD1L in liver cancer and control tissue was tested by Oncomine database (*P*-value <0.01, FC = 2.887). (**C,D**) IHC analysis of LIHC and normal tissues. **P<0.01, ***P<0.001

In order to implore the MTHFD1L expression levels in LIHC and normal liver tissue samples, the expression level of MTHFD1L in tumors and normal tissue of LIHC were analyzed on TCGA and Oncomine databases. We download the data of LIHC and control samples from the TCGA database, which include 370 LIHC and 51 normal samples. The result showed that MTHFD1L was up-regulated in liver cancer compared with control sample (Figure 1A). Also, a total of 35 tumors and 10 controls from the Oncomine database confirmed the high expression of MTHFD1L, as shown in Figure 1B. To further determine the above results, IHC method was used to detect the expression of MTHFD1L in 15 liver cancer tissues and the paired adjacent normal tissues. We found that MTHFD1L expression was up-regulated in LIHC group (Figure 1C,D).

### The prognostic value of MTHFD1L in LIHC

**Figure 2:**
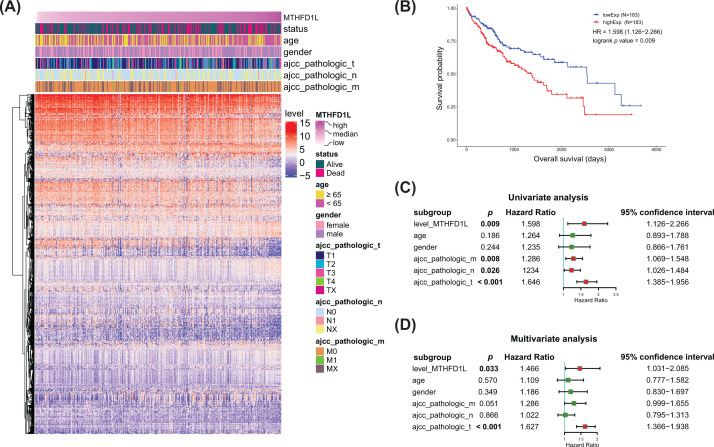
The relationship between MTHFD1L expression of liver cancer and clinicopathological characteristics in TCGA database (**A**) Heatmap of differentially expressed genes in 366 samples and correlation strength of MTHFD1L expression level with different clinicopathological features. (**B**) Survival curves of MTHFD1L high-expression group and low-expression group in 366 samples in TCGA. (**C**) Univariate Cox regression analysis of the correlation between the expression of MTHFD1L expression and other clinicopathological factors in liver cancer. (**D**) Multivariate Cox analysis of the relationship of MTHFD1L expression level and other clinicopathological characteristics in liver cancer.

We next investigated the associations between various clinical parameters and expression of MTHFD1L. The complete clinical information of the LIHC patients included in our study is shown in Table 1. The clinical features include age, gender, vital status, ajcc pathologic m, n and t status, in which we found the low-expression of MTHFD1L group and high-expression of MTHFD1L group were significantly associated with vital status (alive and dead, *P*-value = 0.038). Next, Kaplan–Meier analysis was explored whether the expression of MTHFD1L has an effect in LIHC OS; the result showed that high expression of MTHFD1L was associated with poor OS (Figure 2B). Additionally, heatmap visually showed the relationship between clinical characteristics with MTHFD1L expression, and the clustering of differentially expressed genes in the high- and low-expression MTHFD1L groups (Figure 2A).

**Table 1:** Correlation between the expression level of MTHFD1L and clinical characteristics

Characteristics	Number of cases (%)	Expression of MTHFD1L		*P*-value
		Low (number of cases)	High (number of cases)	
**Age**				
<65	220 (60.1)	105	115	0.286
≥65	146 (39.9)	78	68	
**Gender**				
male	246 (67.2)	121	125	0.656
female	120 (32.8)	62	58	
**Vital_status**				
Alive	237 (64.8)	128	109	0.038
Dead	129 (35.2)	55	74	
**ajcc_pathologic_m**				
M0	264 (72.1)	129	135	0.777
M1	4 (1.1)	2	2	
MX	98 (26.8)	52	46	
**ajcc_pathologic_n**				
N0	251 (68.6)	125	126	0.994
N1	4 (1.1)	2	2	
NX	111 (30.3)	56	55	
**ajcc_pathologic_t**				
T1	180 (49.2)	102	78	0.076
T2	92 (25.1)	41	51	
T3	80 (21.9)	35	45	
T4	13 (3.6)	4	9	
TX	1 (0.3)	1	0	

Then, univariate and multivariate Cox regression analyses were performed to research the correlation between OS and clinical characteristics. The univariate Cox regression demonstrated that the ajcc pathologic m, n and t status and level of MTHFD1L were identified as independent risk factors effectively to affect the OS of LIHC patients (Figure 2C). In addition, multivariate Cox proportional hazards regression analysis was performed to determine which clinical features could contribute to the OS. As a result, ajcc pathologic t status and level of MTHFD1L were significantly connected with OS as shown in Figure 2D. These results reveal that the expression of MTHFD1L can be used as an independent clinical factor to affect the OS and prognosis of LIHC patients.

### Immune infiltration of MTHFD1L in LIHC

**Figure 3:**
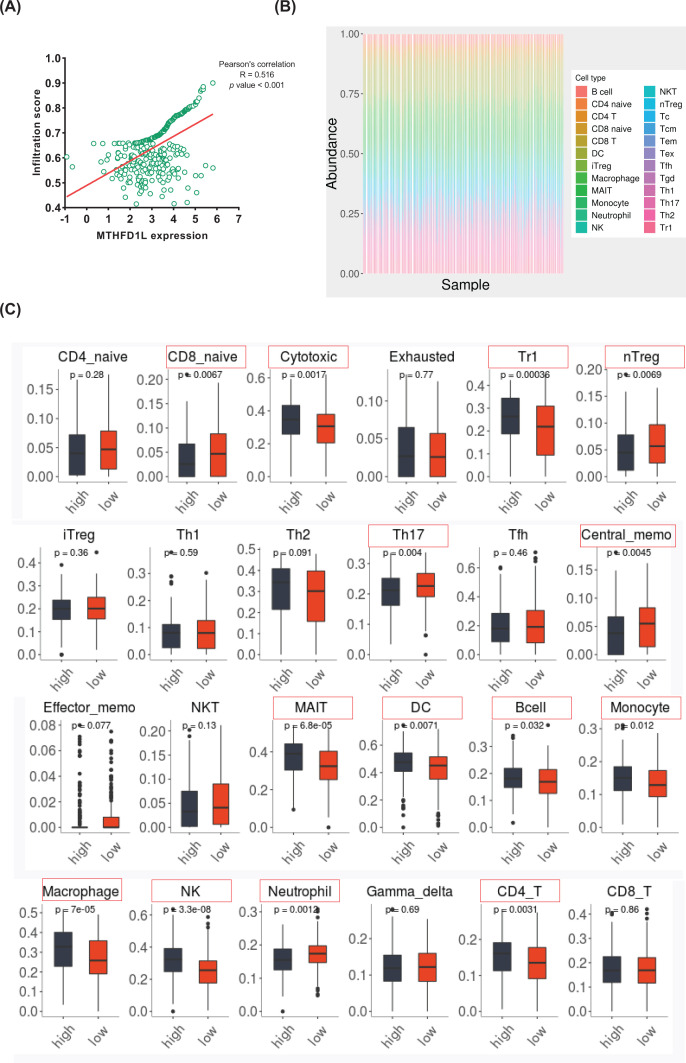
The correlation of MTHFD1L and immune infiltration in liver cancer (**A**) The analysis of MTHFD1L expression level immune infiltration in cancer (*P*-value <0.01, R = 0.516). (**B**) Immune cells estimated by ImmuCellAI in 366 liver cancer. (**C**) The comparison of immune cells with high and low expression of MTHFD1L groups.

To investigate the effect of MTHFD1L expression on different immune cell types in the LIHC microenvironment, the correlation between MTHFD1L expression and immune score was shown in Figure 3A, the MTHFD1L expression was significantly correlated with immune scores (R = 0.516, *P*-value <0.001). To gain a comprehensive analysis of immune cells in LIHC, the ImmuCellAI database was utilized to calculate the composition of 24 immune cells in 366 LIHC sample. As was shown in Figure 3B, the immune cells varied significantly among different samples. Therefore, we compared the level of immune cells in the high expression MTHFD1L group and low expression group in the above study. CD8 navie, nTreg, Th17, central memo and neutrophil were negatively correlated with MTHFD1L expression. Cytotoxic, Tr1, Th2, MAIT, dendritic cell (DC), B cell, monocyte, macrophage, NK and CD4 T were positively correlated with MTHFD1L expression. In Figure 3C, immune cells with differences are marked with red squares (*P*-value <0.01).

### Identification of immune cells closely related to MTHFD1L expression

Subsequently, we studied the correlation between MTHFD1L expression and the immune cells abundance that are different in Figure 3C based on the Pearson’s correlation coefficient. We found that the high expression of MTHFD1L in Table 2 was positively correlated with the expression of DC, Macrophage and NK cells, but negatively correlated with the expression of CD8 naive cells, and was not significantly correlated with other immune cells (*P*-value <0.01).

**Table 2:** Correlation between MTHFD1L and the abundance of immune cells

Cell types	Expression of MTHFD1L	
	Pearson’s correlation	*P*-value
CD8 naive	−0.213	<0.001
Cytotoxic	0.167	0.001
Tr1	0.179	0.001
nTreg	−0.158	0.002
Th17	−0.163	0.002
Central memo	−0.089	0.087
MAIT	0.15	0.004
DC	0.219	<0.001
B cell	0.149	0.004
Monocyte	0.175	0.001
Macrophage	0.261	<0.001
NK	0.213	<0.001
Neutrophil	−0.181	0.001
CD4 T	0.096	0.065

**Figure 4:**
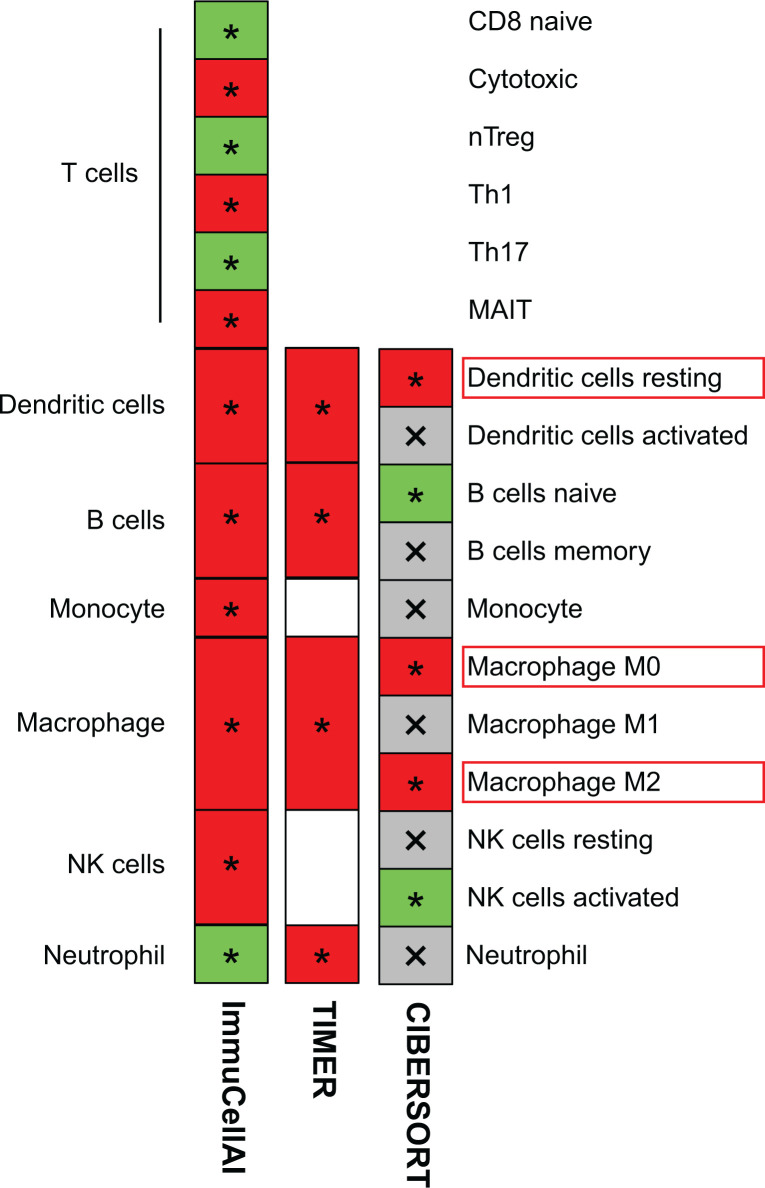
The correlation between MTHFD1L and immune cells Three databases CIBERSORT, TIMER and ImmuneCellAI were used to validate the correlation between MTHFD1L and immune cells (*P<0.05, red means up-regulated and green means down-regulated).

To verify immune cells in LIHC are specifically affected by MTHFD1L expression, we combined three databases CIBERSORT, TIMER and ImmuneCellAI to comprehensively analyze the correlation between various immune cell subtypes and MTHFD1L expression. We found that DCs resting, macrophage M0 and macrophage M2 had a positive correlation with MTHFD1L (Figure 4).

## Discussion

LIHC is one of the most common types of cancer with poor prognosis and high mortality. In recent years, its prevalence has continued to increase [28,29]. Many previous studies have provided information on liver cancer biomarkers [30–32], but so far, the biomarkers that can predict the survival of LIHC patients is still not efficient. Numerous previous studies have reported that MTHFD1L has formyltetrahydrofolate synthase activity and participates in the folic acid cycle, and plays a vital role in supporting tumor growth [14,33,34]. MTHFD1L is overexpressed in multiple solid cancers, such as colorectal cancer [14], tongue squamous cell carcinoma [35], esophageal squamous cell carcinoma [36]. In view of this, we explored the possibility of MTHFD1L as a potential biomarker of LIHC prognosis, and analyzed the interaction between tumor immune infiltration and MTHFD1L expression in LIHC.

Correlation analysis suggested that differences of vital status between high and low expression MTHFD1L groups of LIHC were mainly involved in alive and dead, this illustrates that the expression of MTHFD1L has prognostic value to some extent. An early study identifies MTHFD1L in the folate cycle as an important metabolic pathway in liver cancer cells with the potential for therapeutic [11]. The following Kaplan–Meier analysis also confirmed this view, and the high-expression MTHFD1L group of LIHC had a poor OS curve. Cox regression was performed to identify which clinicopathological features have a significant impact on OS. Considering that the univariate Cox model is insufficient to determine the factors that affect OS, we first used the univariate Cox model to screen for clinical factors related to OS, and the multivariate Cox model to improve the predictive performance of prognostic indicators. Finally, it was found that level of MTHFD1L and ajcc pathologic t status were two risk factors affecting the survival of LIHC patients.

Immune cell infiltration in TME is considered to play an important role in the biological behavior of many kinds of tumors. Until now, many studies have focused on immune landscape and its further clinical application [37–39]. As an essential component of TME, DCs were regarded to be antigen-presenting cells that maintain immune responses. DCs could migrate to tumor tissues through blood circulation in the TME. At different stages of tumor progression, DCs may have different functions, which may be immunostimulatory factors or immunosuppressive factors [40–42]. Macrophages can mediate the proliferation, migration and invasion of tumor cells through a variety of molecular mechanisms, and macrophages infiltration was closely correlated with cancer patient prognosis and therapeutic effect [43,44]. Studies have reported that M0 macrophages can internalize lung tumor-derived exosomes and differentiate into M2 phenotype, while M2 macrophages are characterized by pro-tumorigenic properties [45,46]. Our research provided a comprehensive analysis on the TIICs in LIHC and suggested that M2 macrophages and DCs were associated with the MTHFD1L in LIHC tissue. The analysis results indicated that high expression of MTHFD1L could increase levels of M0 macrophages, M2 macrophages and DCs in the TME of LIHC, which should be helpful for clinical surveillance and treatment of LIHC.

To sum up, MTFHD1L is highly expressed in LIHC through our bioinformatics analyses. Increased MTHFD1L expression correlates with poor prognosis and increased immune infiltration levels in DCs, M0 macrophages and M2 macrophages. Therefore, MTHFD1L may plays an important role in immune cell infiltration and as a prognostic biomarker in LIHC patients.

## Data Availability

All the data will be provided on reasonable request from the corresponding author.

## Abbreviations

CI: Confidence Interval
DC: dendritic cell
HR: Hazard Ratio
IHC: immunohistochemistry
ImmuCellAI: Immune Cell Abundance Identifier
LIHC: liver hepatocellular carcinoma
MAIT: mucosal-associated invariant T
MTHFD1L: methylenetetrahydrofolate dehydrogenase 1-like
NK: natural killer cell
nTreg: naturally-occurring regulatory T cells
OS: overall survival
RNA-Seq: RNA-Sequence
TCGA: The Cancer Genome Atlas
Th17: T helper cell 17
TIIC: tumor-infiltrating immune cell
TME: tumor microenvironment
Tr1: type 1 T regulatory cells
